# Unbalance between Pyridine Nucleotide Cofactors in The SOD1 Deficient Yeast *Saccharomyces cerevisiae* Causes Hypersensitivity to Alcohols and Aldehydes

**DOI:** 10.3390/ijms24010659

**Published:** 2022-12-30

**Authors:** Magdalena Kwolek-Mirek, Sabina Bednarska, Aleksandra Dubicka-Lisowska, Roman Maslanka, Renata Zadrag-Tecza, Pawel Kaszycki

**Affiliations:** 1 Department of Biology, Institute of Biology and Biotechnology, College of Natural Sciences, University of Rzeszow, 35-601 Rzeszow, Poland; 2 Department of Plant Biology and Biotechnology, Faculty of Biotechnology and Horticulture, University of Agriculture in Krakow, 31-425 Krakow, Poland

**Keywords:** alcohol dehydrogenase, aldehyde dehydrogenase, alcohols and aldehydes toxicity, allyl alcohol, acrolein, nicotinamide adenine dinucleotide phosphate generation, pyridine nucleotide cofactors, redox homeostasis, superoxide dismutase 1

## Abstract

Alcohol and aldehyde dehydrogenases are especially relevant enzymes involved in metabolic and detoxification reactions that occur in living cells. The comparison between the gene expression, protein content, and enzymatic activities of cytosolic alcohol and aldehyde dehydrogenases of the wild-type strain and the Δ*sod1* mutant lacking superoxide dismutase 1, which is hypersensitive to alcohols and aldehydes, shows that the activity of these enzymes is significantly higher in the Δ*sod1* mutant, but this is not a mere consequence of differences in the enzymatic protein content nor in the expression levels of genes. The analysis of the NAD(H) and NADP(H) content showed that the higher activity of alcohol and aldehyde dehydrogenases in the Δ*sod1* mutant could be a result of the increased availability of pyridine nucleotide cofactors. The higher level of NAD^+^ in the Δ*sod1* mutant is not related to the higher level of tryptophan; in turn, a higher generation of NADPH is associated with the upregulation of the pentose phosphate pathway. It is concluded that the increased sensitivity of the Δ*sod1* mutant to alcohols and aldehydes is not only a result of the disorder of redox homeostasis caused by the induction of oxidative stress but also a consequence of the unbalance between pyridine nucleotide cofactors.

## 1. Introduction

Alcohol dehydrogenases (ADHs) and aldehyde dehydrogenases (ALDs) are the enzymes involved in cellular metabolism as well as in the detoxification of alcohols, and aldehydes accumulate under stress conditions. ADHs are NAD-dependent oxidoreductases that catalyze the reversible oxidation of primary and secondary alcohols into aldehydes and ketones, respectively. The yeast alcohol dehydrogenase family (Y-ADH) belongs to the medium-chain superfamily, which consists of enzymes with a subunit size of *c.* 350 amino acid residues, either dimeric or tetrameric, with two domains in each subunit: one catalytic and one binding a coenzyme. The catalytic domain contains a ‘catalytic’ zinc atom which is responsible for alcohol binding and a ‘structural’ zinc atom which plays a conformational role, probably through the stabilization of the tertiary structure of the enzyme [1,2,3]. There are five genes encoding classical Y-ADHs (*ADH1*-*ADH5*) involved in ethanol metabolism in *Saccharomyces cerevisiae*. Three of these enzymes, Adh1p, Adh2p, and Adh5p, are cytosolic proteins, whereas Adh3p and Adh4p are located in the mitochondria [2]. Adh1p, Adh3p, Adh4p, and Adh5p reduce acetaldehyde to ethanol, while Adh2p mainly catalyzes the reverse reaction and oxidation of ethanol to acetaldehyde. Adh1p is a constitutive enzyme, but its biosynthesis can be repressed during cell culture at the stationary phase or upon transfer to non-fermentable carbon sources [3,4]. Adh1p is primarily responsible for the production of ethanol during anaerobic growth (fermentative carbon metabolism). In turn, Adh2p is a major ethanol oxidizer and is found only in the case of aerobically grown yeast cells (respiratory carbon metabolism). Growth in the presence of glucose inhibits the expression of *ADH2* and *ADH3* genes [5]. However, the repression of *ADH3* is not as severe as that of *ADH2* [6,7], and *ADH3* is derepressed when glucose is depleted from the medium. Adh4p is a protein that normally occurs at low concentrations in laboratory strains [8] and is considered important for yeast survival at relatively less favorable growth conditions [9]. Under the conditions where Adh1p is nonfunctional, the spontaneous chromosomal amplification of *ADH4* was found to be able to rescue the mutant phenotype [10]. In turn, the Adh5p activity was revealed only in a double mutant devoid of *ADH1* and *ADH3* [11].

Aldehyde dehydrogenases (ALDs) are oxidoreductases that catalyze the reversible oxidation of aldehydes to acids associated with the reduction in NAD^+^ or NADP^+^. ALDs play a crucial role in the conversion of acetaldehyde to acetate, which can then be used for acetyl-CoA synthesis or excreted outside the cell. There are five genes encoding ALDs (*ALD2*-*ALD6*) that are involved in ethanol metabolism in *S. cerevisiae*. Three of these enzymes, Ald2p, Ald3p, and Ald6p, are cytosolic proteins, whereas Ald4p and Ald5p are located in the mitochondria. Ald2p and Ald3p oxidize ethanol to acetaldehyde, while Ald4p, Ald5p, and Ald6p are required for the conversion of acetaldehyde to acetate. It is worth emphasizing that Ald2p and Ald3p can use NAD^+^ as the preferred cofactor, while Ald5p and Ald6p utilize mainly NADP^+^. Ald4p, in turn, is dependent on both NAD^+^ and NADP^+^ [12,13,14,15]. *ALD2* and *ALD3* genes are induced in response to ethanol and become repressed by glucose. Moreover, *ALD3* is induced by a variety of stress factors, including osmotic stress, heat shock, glucose exhaustion, oxidative stress, and drugs. In turn, *ALD2* is induced only by osmotic stress and glucose exhaustion [15]. The *ALD4* gene encoding the major mitochondrial aldehyde dehydrogenase is repressed by glucose. Ald4p and Ald5p are activated by K^+^, while Ald6p is activated by Mg^2+^ [13]. Ald5p and Ald6p isoforms are expressed constitutively and play a major role in acetate formation during the anaerobic growth of yeast cells on glucose [16].

Our previous studies have shown that the mutant cells lacking superoxide dismutase 1 (SOD1) were hypersensitive to alcohols and aldehydes. The increased sensitivity of the mutant yeast was explained by a disorder of redox homeostasis caused by the induction of oxidative stress in the cells [17,18,19]. In this study, additional key factors are shown to contribute to the observed increased sensitivity of the Δ*sod1* mutant to alcohols and aldehydes. The experiments were based upon the use of allyl alcohol, the metabolic precursor of acrolein, pyrazole, an inhibitor of alcohol dehydrogenase and disulfiram, and an inhibitor of aldehyde dehydrogenase used to modulate the level of aldehyde in the cells. The gene expression, protein content, and enzymatic activities of cytosolic alcohol and aldehyde dehydrogenases were investigated. The levels of pyridine nucleotide cofactors and tryptophan, as well as the expression, synthesis, and activity of the selected enzymes of the pentose phosphate pathway (PP pathway), were also analyzed.

## 2. Results

### 2.1. Inhibition of Alcohol Dehydrogenase and Aldehyde Dehydrogenase Activities Influences the Toxicity of Allyl Alcohol and Acrolein in Yeast

**Figure 1:**
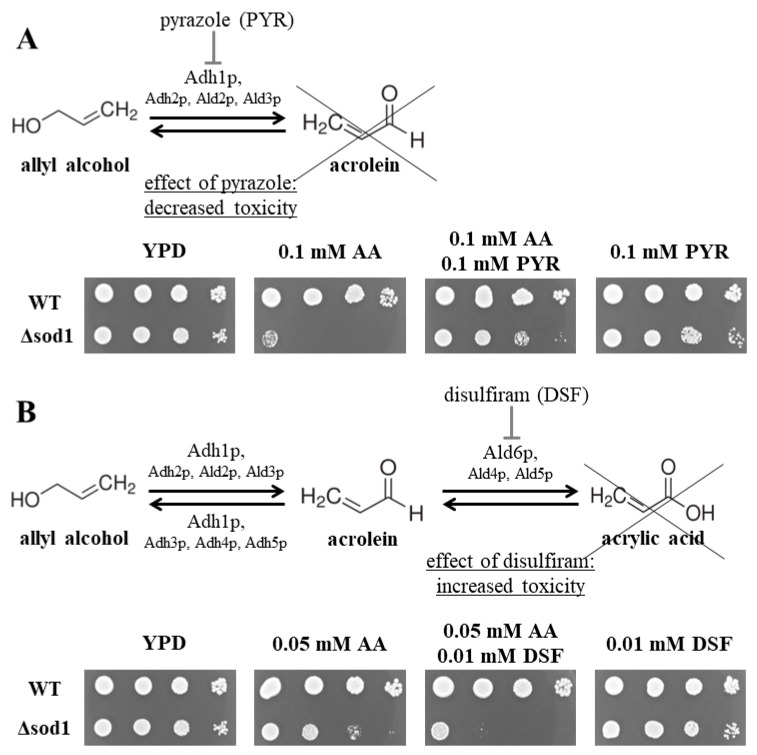
Metabolism of allyl alcohol (AA) and acrolein (Acr) in the *Saccharomyces cerevisiae* yeast. The wild-type (WT) strain and Δ*sod1* mutant were cultivated in the YPD liquid medium for 16 h, diluted serially (1:10), and suspensions were spotted onto solid YPD plates containing allyl alcohol (0.05- or 0.1 mM AA) and 0.1 mM pyrazole (PYR) (**A**) or 0.01 mM disulfiram (DSF) (**B**). Successive spots contained 50,000, 5000, 500, and 50 cells. Plates were cultivated at 28 °C and colony growth was recorded after 48 h.

Alcohol and aldehyde dehydrogenases are the main enzymes responsible for allyl alcohol (AA) metabolism in the yeast *S. cerevisiae*. Alcohol dehydrogenase (ADH) oxidizes allyl alcohol to acrolein (Acr)—a highly reactive and toxic aldehyde [20], and the exposure of the yeast to AA leads to a considerable increase in acrolein content in the cells [17]. A total of 0.1 mM AA caused a significant inhibition of the Δ*sod1* mutant growth without negative effects on the WT strain (Figure 1A). Pyrazole (PYR), which is a competitive inhibitor of ADH [21], binds reversibly to the active center of the enzyme preventing AA binding and thus preventing Acr formation. A total of 0.1 mM PYR did not affect the growth of both tested strains (Figure 1A). In turn, the addition of 0.1 mM PYR to the medium with 0.1 mM AA resulted in a decrease in AA toxicity for the case of Δ*sod1* mutant (Figure 1A). The growth of the WT strain was the same as that under control conditions (YPD medium without additives) (Figure 1A).

Next, it was examined whether an increased concentration of acrolein (formed intracellularly from allyl alcohol) could inhibit the growth of yeast cells any stronger. For this purpose, 0.05 mM AA, which caused only a slight growth inhibition in the case of the Δ*sod1* mutant (Figure 1B), and disulfiram (DSF), an inhibitor of aldehyde dehydrogenase [22], were used. DSF prevents the conversion of Acr to the less toxic acrylic acid, thus increasing the concentration of the aldehyde in the cells. A total of 0.01 mM DSF did not affect the growth of both tested strains (Figure 1B). In turn, the addition of 0.01 mM DSF to the medium with 0.05 mM AA resulted in the increased toxicity of AA in the Δ*sod1* mutant (Figure 1B). The growth of the treated WT strain was the same as that under the control conditions (YPD medium without additives) (Figure 1B). These results show that the inhibition of ADH and ALD activities in the presence of specific inhibitors had an influence on the toxicity of AA and Acr in the yeast. The obtained data also confirm our previous reports showing that the toxicity of AA was not a result of its direct action but rather of intracellular Acr formation [17,18].

### 2.2. Higher Activity of Alcohol Dehydrogenase and Aldehyde Dehydrogenase in the Δsod1 Mutant Cells Determines the Yeast’s Hypersensitivity to Alcohols and Aldehydes

**Figure 2:**
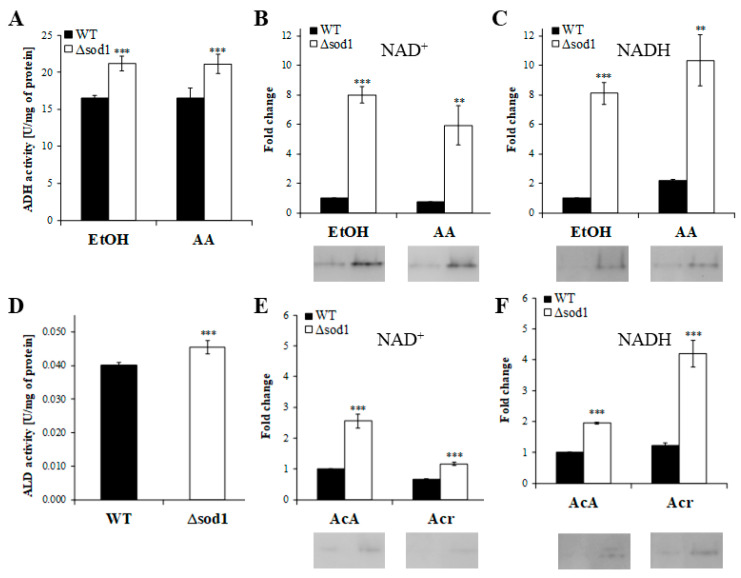
Activity of alcohol dehydrogenase (ADH) and aldehyde dehydrogenase (ALD) in the whole-cell protein extracts prepared for the wild-type (WT) strain and Δ*sod1* mutant. ADH activity was determined by measuring the rate of NAD^+^ reduction at 340 nm with ethanol (EtOH) and allyl alcohol (AA) (**A**) and by staining in the gel after native PAGE electrophoresis with ethanol (EtOH) and allyl alcohol (AA) as substrates as well as with NAD^+^ (**B**) and NADH (**C**) as reaction cofactors. ALD activity was determined with 6-methoxy-2-naphthaldehyde as a substrate in the presence of NAD^+^ (**D**). Fluorescence signal increase was recorded at λex = 310 nm and λem = 360 nm. ALD activity was determined by also staining in the gel after native PAGE electrophoresis with acetaldehyde (AcA) and acrolein (Acr) as substrates as well as with NAD^+^ (**E**) and NADH (**F**) as reaction cofactors. The results are presented as mean ± SD from four independent experiments in each case. ** *p* < 0.01, *** *p* < 0.001 as compared to the results for the WT strain.

Alcohol dehydrogenase activity in whole-cell protein extracts was determined by measuring the rate of NAD^+^ reduction in the presence of allyl alcohol as a substrate at λ = 340 nm. For comparison, the ADH activity was also assessed with ethanol. It was shown that the activity of ADH was 28% higher in the Δ*sod1* mutant compared to the WT strain for the case of both tested alcohols (Figure 2A). Even higher ADH activity in the Δ*sod1* mutant was confirmed by enzymatic staining in a polyacrylamide gel (Figure 2B,C). It was shown that the activity of ADH, when NAD^+^ was used as a cofactor, was eight times higher in the Δ*sod1* mutant when compared to the WT strain for both tested alcohols (Figure 2B). In turn, when we used NADH as a cofactor of this reaction, the ADH activity was eight and five times higher for ethanol and allyl alcohol, respectively (Figure 2C). On the other hand, the ALD activity was determined with 6-methoxy-2-naphthaldehyde (MONAL-62) and acetaldehyde as a substrate. It was shown that the activity of ALD was, respectively, 13% (Figure 2D) and 22% (data not shown) higher in the Δ*sod1* mutant compared to the WT strain. The above results were again confirmed by enzymatic staining in a polyacrylamide gel (Figure 2E,F). It was shown that the activity of ALD, when NAD^+^ was used as a cofactor, was 2.5 and 1.8-times higher in the Δ*sod1* mutant compared to the WT strain for acetaldehyde and acrolein, respectively (Figure 2E). In turn, when NADH was used as a cofactor of this reaction, the ALD activity was 2 and 3.4 times higher for acetaldehyde and acrolein, respectively (Figure 2F). The presented results show that alcohol dehydrogenase and aldehyde dehydrogenase activities are significantly higher in the Δ*sod1* mutant than in the WT strain, which may cause hypersensitivity to alcohols and aldehydes of the former strain.

### 2.3. Changes in the Expression of Genes Encoding Alcohol and Aldehyde Dehydrogenases Do Not Affect the Content of These Enzymes in the Δsod1 Mutant Cells

**Figure 3:**
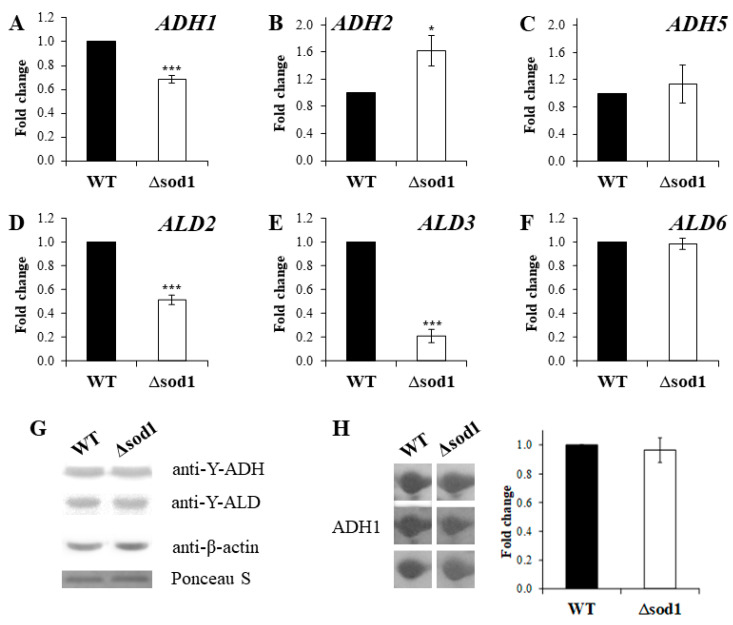
*ADH* and *ALD* gene expression and ADH and ALD protein content in the wild-type (WT) strain and Δ*sod1* mutant. *ADH* (**A**–**C**) and *ALD* (**D**–**F**) gene expression were performed by qPCR assay with TaqMan probes. The relative gene expression was calculated with a comparative C_T_ method: the −ΔΔC_T_ method for comparison of individual gene expression between the WT and Δ*sod1* mutant. The results are presented as mean ± SD from three independent experiments. * *p* < 0.05, *** *p* < 0.001 as compared to the results for the WT strain. The ADH and ALD proteins were detected by the immunoblotting assay with the primary antibodies: anti-yeast alcohol dehydrogenase (1:5000), anti-yeast aldehyde dehydrogenase (1:5000), and anti-β-actin (1:5000) with the horseradish peroxidase-conjugated secondary antibodies (1:10,000 or 1:10,000) and a chemiluminescent substrate (**G**). Protein identification was performed by two-dimensional gel electrophoresis and then by liquid chromatography coupled to the mass spectrometer. The picture represents the results from three independent experiments (**H**).

In the yeast *S. cerevisiae*, there are three genes coding for cytosolic isoenzymes, both in the case of alcohol dehydrogenase (*ADH1*, *ADH2*, *ADH5*) and aldehyde dehydrogenase (*ALD2*, *ALD3*, *ALD6*). The level of expression for individual genes using the −ΔΔC_T_ method for comparison with the WT strain and the Δ*sod1* mutant was calculated (Figure 3A–F). It was shown that the expression of *ADH1*, *ALD2,* and *ALD3* genes was down-regulated in the Δ*sod1* mutant (Figure 3A,D,E). The level of expression was approx. 1.4, 1.9, and 4.7 times lower for the case of *ADH1*, *ALD2,* and *ALD3* genes, respectively (Figure 3A,D,E). There was no difference in the expression of *ADH5* and *ALD6* genes between both tested strains (Figure 3C,F). In turn, the expression of the *ADH2* gene was up-regulated in the Δ*sod1* mutant in comparison to the WT strain (Figure 3B). Furthermore, the relative expression of these genes (−ΔC_T_) in the same strains was calculated (Figure S1). It can be seen that, during the growth of the yeast in the medium with glucose, the highest level of expression was determined for the case of *ADH1* and *ALD6* genes. However, the *ALD6* gene expression level was about 4.2 times lower when compared to the *ADH1* gene (Figure S1). On the other hand, the lowest level of expression (almost no expression) was noted for the case of the *ADH2* and *ALD2* genes (Figure S1). Due to the fact that some changes in the *ADH* and *ALD* gene expression levels were observed between the tested strains, we decided to check if any changes in the content of the ADH and ALD proteins occurred. In order to determine the ADH and ALD protein content, the Western Blot method with anti-Y-ADH and anti-Y-ALD antibodies was employed. It was shown that there were no differences in the level of these proteins in both tested strains (Figure 3G). In addition, the content of the ADH1 protein, the main alcohol dehydrogenase isoenzyme in the yeast, was compared with the 2-DE gel electrophoresis. The quantitative analysis of the respective gel spots clearly showed that the accumulation of the ADH1 protein was almost the same in both tested strains (Figure 3H). Such a result proves that the higher activity of ADH and ALD in the Δ*sod1* mutant compared to the WT strain (Figure 2) is not a mere consequence of differences in the accumulation of these enzymes (Figure 3G,H) nor in the expression levels of the genes coding for these proteins (Figure 3A,C–F). The upregulation of *ADH2* gene expression (Figure 3B) cannot give an explanation of the above phenomenon, perhaps due to the low level of relative expression for this gene in the yeast cells (Figure S1) during growth in the medium with glucose.

### 2.4. NAD(H) and NADP(H) Content Determines the Activity of Alcohol Dehydrogenase and Aldehyde Dehydrogenase in the Yeast

**Figure 4:**
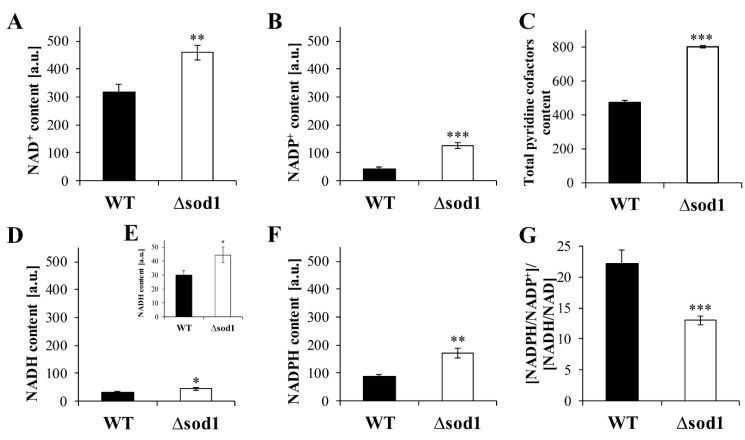
Individual (**A**,**B**,**D**–**F**) and total (**C**) pyridine nucleotide cofactors content and [NADPH/NADP^+^]/[NADH/NAD] ratio (**G**) in the wild-type strain (WT) and Δ*sod1* mutant. The content of pyridine nucleotide cofactors was assessed with NAD/NADH-Glo and NADP/NADPH-Glo assays. Data are presented as mean ± SD from three independent experiments. * *p* < 0.05, ** *p* < 0.01, *** *p* < 0.001 as compared to the WT strain.

Alcohol dehydrogenase and aldehyde dehydrogenase are oxidoreductases that utilize mainly NAD^+^ or NADP^+^ as cofactors. The availability of pyridine nucleotide cofactors can affect the activity of enzymes and, thus, the metabolism and detoxification reactions of alcohols and aldehydes in the cells. It was shown that the content of both individual and total pyridine nucleotide cofactors was significantly higher in the Δ*sod1* mutant compared to the WT strain (Figure 4A–F). The level of NAD^+^ and NADH was 1.4 times higher in the Δ*sod1* mutant than in the WT strain (Figure 4A,D,E). In turn, the difference observed for the case of NADP^+^ and NADPH was 3.2 and two-fold, respectively (Figure 4B,F). These results suggest that the higher activity of ADH and ALD in the Δ*sod1* when compared to the WT strain (Figure 2), can be a result of the increased availability of pyridine nucleotide cofactors in the Δ*sod1* mutant cells (Figure 4A–F). On the other hand, the (NADPH/NADP^+^)/(NADH/NAD^+^) ratio was substantially decreased in the Δ*sod1* mutant in comparison to the WT strain (Figure 4G). These results demonstrate that the lack of the SOD1 enzyme leads to a disturbance in intracellular redox homeostasis.

### 2.5. Increased Generation of Pyridine Nucleotide Cofactors Causes Higher Activity of Glucose-6-Phosphate Dehydrogenase and 6-Phosphogluconate Dehydrogenase in the Δsod1 Mutant

**Figure 5:**
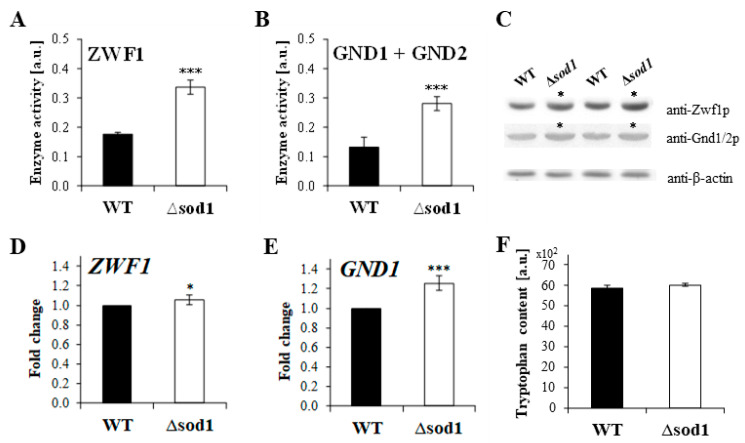
Activity, gene expression, and protein detection of glucose-6-phosphate dehydrogenase (ZWF1) and 6-phosphogluconate dehydrogenase (GND) in the wild-type (WT) strain and Δ*sod1* mutant. The activity of glucose-6-phosphate dehydrogenase (ZWF1) was calculated by subtracting the activity of GND1 and GND2 from the total enzyme activity (**A**). The total activity of the PP pathway dehydrogenases (sum of both ZWF1 and GND1/GND2 activities) was determined with D-glucose-6-phosphate and 6-phosphogluconate as substrates and 6-phosphogluconate dehydrogenase (GND1 and GND2) activity was determined with 6-phosphogluconate, in the presence of NADP^+^ (**B**). The ZWF1 and GND1/2 proteins were detected by immunoblotting assay with the primary antibodies: anti-glucose-6-phosphate dehydrogenase (1:2000), anti-6-phosphogluconate dehydrogenase (1:2000), and anti-β-actin (1:5000) and with the horseradish peroxidase-conjugated secondary antibodies (1:10,000 or 1:10,000) with a chemiluminescent substrate (**C**). *ZWF1* (**D**) and *GND1* (**E**) gene expression was performed by qPCR assay with TaqMan probes. The relative gene expression was calculated with a comparative C_T_ method: the −ΔΔC_T_ method for comparison of individual gene expression between the WT and Δ*sod1* mutant. The results are presented as mean ± SD from three independent experiments. * *p* < 0.05, *** *p* < 0.001 as compared to the results for the WT strain. The tryptophan content in the cell extracts was determined by fluorimetric measurements at λex = 290 nm and λem = 325 nm. The results are presented as mean ± SD from three independent experiments.

The activity of glucose-6-phosphate dehydrogenase (ZWF1) and 6-phosphogluconate dehydrogenase (GND1 and GND2) is responsible for NADPH formation in the PP pathway. It was shown that the activity of these enzymes was increased in the Δ*sod1* mutant relative to the WT strain (Figure 5A,B). The higher activity of these enzymes may result from the upregulation of *ZWF1* and *GND1* gene expression (Figure 5D,E) and the higher content of Zwf1p and Gnd1/Gnd2p proteins (Figure 5C), as well as a higher level of NADP^+^ (Figure 4B) in the Δ*sod1* mutant than in the WT strain. The increased activities of the PP pathway dehydrogenases and a higher level of their cofactor (NADP^+^) bring higher levels of NADPH in the Δ*sod1* mutant cells (Figure 4F). In addition, the tested strains did not differ in the cellular levels of tryptophan (Figure 5F) which could be used for the de novo production of NAD^+^ and can then be further converted to other pyridine nucleotide cofactors. The results demonstrate that the higher generation of NADPH in the Δ*sod1* mutant is associated with the upregulation of the PP pathway.

## 3. Discussion

Pyridine nucleotide redox couples are essential for maintaining cellular redox homeostasis and modulating numerous biological events, including cellular metabolism. The NAD^+^/NADH couple is primarily responsible for cellular catabolism reactions and energy generation and plays an important role in signaling pathways. In the yeast *Saccharomyces cerevisiae*, NAD^+^ can be synthesized de novo from tryptophan by the kynurenine pathway or salvaged from intermediates and small precursors such as nicotinic acid, nicotinamide, and nicotinamide riboside. Yeast cells release and re-uptake these precursors from culture media [23,24,25,26]. In this study, it was shown that NAD^+^ content in the Δ*sod1* mutant was higher than in the WT strain (Figure 4A), but at the same time, the tryptophan content was at a similar level (Figure 5F). Higher levels of NAD^+^ in the case of the Δ*sod1* mutant may be partly explained by the incomplete repression of respiratory metabolism in the presence of glucose in the cells of this mutant. Reddi and Culotta showed that oxygen, glucose, and the SOD1 enzyme cooperated to maintain the stability of Yck1p and Yck2p: two CK1γ casein kinases for respiration repression. The lack of SOD1 affected the glucose and amino acid sensing pathways that required Yck1p and Yck2p [27]. In our earlier studies, we proved that the NAD^+^ content was higher in the yeast that conducted respiratory metabolism than in the cells performing fermentation [28]. Another possible explanation for the high NAD^+^ content in the Δ*sod1* mutant is its lower intracellular conversion and utilization. NAD^+^ is consumed upon the activity of several enzymes, including sirtuins (SIRTs) and NAD^+^-dependent histone deacetylases, whose activity in yeast and other organisms is connected with the regulation of lifespan. The possibility of the lower consumption of NAD^+^ by SIRTs arises from the following facts: (i) cysteines in the catalytic domain in yeast Sir2p upon oxidative and disulfide stress are S-glutathionylated, which results in enzyme inactivation [29]; (ii) there exists a correlation between the expression of *SOD1* and *SOD2* genes, the activity of superoxide dismutases, the expression of the *SIR2* gene, and the activity of sirtuins [30]; (iii) the depletion of SOD1 increases both intracellular ROS content and sensitivity to conditions and agents which decrease the level of reduced glutathione [18,19]; (iv) the loss of *SOD1* reduces the replicative lifespan of the yeast cells [31].

NAD^+^ is converted to other pyridine nucleotides, which may be critical for maintaining a proper redox state and cellular function under stress conditions. NAD^+^ can be reduced to NADH via dehydrogenases and can be phosphorylated to NADP^+^ via NAD^+^ kinases. In this study, it was shown that the level of both NADH and NADP^+^ in the Δ*sod1* mutant was higher than in the WT strain (Figure 4B,D). In turn, the increased level of NADP^+^ could influence the PP pathway in which glucose-6-phosphate is converted to ribulose-5-phosphate with the simultaneous reduction of two NADP^+^ to NADPH. These reactions are catalyzed by glucose-6-phosphate dehydrogenase (ZWF1) and 6-phosphogluconate dehydrogenase (GND1 and GND2) [32,33]. Here, we reveal that in the yeast cells, there exist correlations between the expression of *ZWF1* and *GND1* genes, the content and activity of glucose-6-phosphate dehydrogenase and 6-phosphogluconate dehydrogenase and the level of NADPH (Figure 4F and Figure 5A–E). The other important source of NADPH is the conversion of acetaldehyde to acetate when catalyzed by aldehyde dehydrogenase 6 (ALD6) [34]. The data provided in this work proves that the total activity of aldehyde dehydrogenase but not the *ALD6* gene expression and ALD protein content is increased in the Δ*sod1* mutant (Figure 2D–F and Figure 3F,G). It is worth mentioning that higher levels of NADPH in the cells deficient in SOD1 can be beneficial because NADPH is used to reduce the oxidized form of glutathione by glutathione reductase and the oxidized form of thioredoxins by thioredoxin reductase [35,36]. Furthermore, NADPH is considered to be a directly operating antioxidant, which is effective in both scavenging free radicals and repairing biomolecule-derived radicals [37,38]. Despite the fact that the total pyridine cofactors content is increased in the Δ*sod1* mutant when compared to the WT strain (Figure 4C), the (NADPH/NADP^+^/NADH/NAD^+^) ratio in the former strain is reduced (Figure 4G). There are many consequences of unbalance between the content of pyridine nucleotide cofactors in the cells, including higher sensitivity to alcohols and aldehydes.

Allyl alcohol is oxidized intracellularly to acrolein by alcohol dehydrogenase [17,20]. High levels of both *ADH1* gene expression (Figure S1) and ADH1 protein synthesis (0.5–1% of the total yeast protein abundance) [4] prove that this is the major isoenzyme of alcohol dehydrogenase present in the yeast. Although Adh1p is mainly responsible for the production of ethanol from acetaldehyde, it may also catalyze (though less efficiently) a reverse reaction with the concomitant reduction in NAD^+^ [2,39]. Allyl alcohol serves as a good substrate for this enzyme [40] which oxidizes it more efficiently than ethanol [41]. Moreover, it was shown that the mutant lacking the *ADH1* gene exhibited an elevated resistance to allyl alcohol [42]. Both the level of NAD^+^ and the NAD^+^/NADH ratio are crucial for metabolic reactions occurring in the cells. On one hand, the correlation between the NAD^+^ content, ADH activity, and the level of acrolein was shown to have consequences in terms of the hypersensitivity of the Δ*sod1* mutant to the acrolein [[17] Figure 1A, Figure 2A,B and Figure 4A of this study]. On the other hand, Wills and Martin have documented that elevated levels of NADH, and thus a decreased NAD^+^/NADH ratio, caused a shift of equilibrium between allyl alcohol and acrolein towards the harmless allyl alcohol [42]. However, Adh1p is five times less active in reducing acrolein than in reducing acetaldehyde [41]. In the present work, we revealed that higher levels of NADH in the Δ*sod1* mutant (which is a result of NAD^+^ reduction) might cause elevated ADH activity; however, this reaction does not lead to allyl alcohol resistance, probably due to its relatively low efficiency (Figure 1A, Figure 2C and Figure 4D). The reaction of the intracellular formation of acrolein from allyl alcohol may be, in theory, also catalyzed by Adh2p, Ald2p, and Ald3p isoenzymes. It was shown that the mutant lacking the *ADH2* gene had increased resistance to allyl alcohol [6], the mutant lacking the *ALD2* gene had increased sensitivity to acrolein [43], and the mutant lacking the *ALD3* gene had increased sensitivity to both ethanol and acetaldehyde [44,45]. At the same time, however, the *ADH2*, *ALD2,* and *ALD3* genes were repressed by glucose [5,14,15]. The described results are in line with our findings. The expression of the above-mentioned genes was kept at a very low level, especially in the case of *ADH2* and *ALD2* (Figure 3B,D–E and Figure S1). It can be concluded that the Adh2p, Ald2p, and Ald3p isoenzymes do not perform an important function in cellular metabolism during yeast growth in the medium supplemented with glucose. Moreover, it is noted that the expression of *ADH1*, *ALD2,* and *ALD3* genes are down-regulated in the case of the Δ*sod1* mutant (Figure 3A,D,E and Figure S1), in which an increase in the total ADH and ALD activity can be observed (Figure 2A–F). The lower expression of these genes may be due to another layer of regulation aimed at maintaining the enzyme activity homeostasis as much as possible. This seems to be extremely important in relation to enzymes whose activity generates very toxic intermediates such as aldehydes.

The major pathway of acrolein detoxification in the cells is the conjunction of acrolein with reduced glutathione [17,18,46]. A rapid decrease in the glutathione content and disturbance of cellular redox homeostasis may be the cause of increased ROS production, especially in the case of the Δ*sod1* mutant [19]. Acrolein may also react with other thiol-containing compounds such as cysteine, 2-mercaptoethanesulfonate, 2,6-dithiopurine [47], and with cellular macromolecules, especially proteins and DNA [18,19,48]. Proteomic studies have shown that ADH is one of the most sensitive proteins to oxidative stress in yeast [49,50]. The zinc-thiolate active site center of this enzyme is particularly susceptible to oxidant attacks, and the oxidation of active site thiols leads to zinc release and correlates with enzyme inactivation. ADH activity may be inhibited by superoxide, peroxynitrite, hypochlorite, hydrogen peroxide, and many others [51,52,53]. The Δ*sod1* mutant has a higher level of superoxide anion compared to the WT strain [19,28], which might cause the reduction in activity or even the partial inactivation of ADH in the cells of the mutant. However, as revealed in this study, due to the higher level of NAD^+^ (Figure 4A), the activity of ADH was also higher in the Δ*sod1* mutant than in the WT strain (Figure 2A–F). To our best knowledge, such an effect has been shown for the first time. The consequences of these changes are the increased intracellular generation of acrolein from allyl alcohol [17], a stronger induction of oxidative stress, higher levels of cellular damage, and finally, hypersensitivity to this aldehyde [[18,19,48]; Figure 1 in this study].

Only unbound acrolein can be enzymatically converted to less toxic metabolites. The main isoenzyme of aldehyde dehydrogenase present in the yeast upon the culture in the medium with glucose was Ald6p (Figure S1). Ald6p converted acetaldehyde to acetic acid with the concomitant reduction in NAD^+^ or NADP^+^. The mutant lacking the *ALD6* gene exhibited a reduced growth rate in the glucose-supplemented medium, was unable to use ethanol as a carbon source [13], and had an increased sensitivity to acetaldehyde [44]. The important role of aldehyde dehydrogenase in the metabolism and detoxification of acrolein was confirmed using disulfiram: an inhibitor of ALD. In this study, it was shown that if disulfiram was added to the medium, the Δ*sod1* mutant sensitivity to allyl alcohol would increase (Figure 1C). This confirms that the toxicity of allyl alcohol strongly depends on the concentration of acrolein in the cells. In spite of the fact that NAD^+^ and NADP^+^ content and ALD activity are higher in the Δ*sod1* mutant than in the WT strain (Figure 2D,E and Figure 4A,B), the conversion of acrolein to acrylic acid does not bring a significant reduction in its toxicity. The explanation may be that the major amount of acrolein reacts with cellular nucleophiles, and only a small part of it undergoes further metabolism. Another possible pathway is the conversion of acrolein to propionaldehyde by the NADPH-dependent oxidoreductase—old yellow enzyme 2 (Oye2p). Trotter et al. have evidenced that mutants lacking *OYE2* but not *OYE3* were sensitive to acrolein, whereas the overexpression of both isoenzymes increased acrolein tolerance [43].

In summary, higher levels of NAD^+^ and the unbalance between pyridine nucleotide cofactors affect the functioning of Δ*sod1* mutant cells inter alia by changing their metabolism, redox balance, and stress response. The study clearly shows that there is a correlation between the NAD^+^ content, ADH, and ALD activities, as well as the level of acrolein (formed intracellularly from allyl alcohol) in the Δ*sod1* mutant. As a consequence, a stronger induction of oxidative stress occurs with higher levels of cellular damage and, finally, the hypersensitivity of the cells to this aldehyde. Although the obtained results are focused mainly on the metabolism and toxicity of allyl alcohol and acrolein in the Δ*sod1* mutant, the demonstrated relationships may be also important in the case of other alcohols and aldehydes.

## 4. Materials and Methods

### 4.1. Chemicals

Allyl alcohol ≥ 99%, CAS number 107-18-6; acrolein 90%, CAS number 107-02-8; pyrazole 98%, CAS number 288-13-1, and disulfiram (tetraethylthiuram disulfide) ≥ 97%, CAS number 97-77-8 were from Aldrich (Sigma-Aldrich, St. Louis, MO, USA).

### 4.2. Yeast Strains and Growth Conditions

The following yeast strains were used: *Saccharomyces cerevisiae* wild-type strain SP4 MATα *leu1 arg4* [54] and Δ*sod1* mutant, isogenic to SP4, MATα *leu1 arg4 sod1::natMX* [55]. The yeasts were grown in the standard liquid YPD medium (1% Yeast Extract, 1% Yeast Bacto-Peptone, 2% glucose) on a rotary shaker at 150 rpm or on solid YPD medium containing 2% agar at the temperature of 28 °C.

### 4.3. Spotting Test

The cells collected from the exponential phase of the culture were centrifuged, washed with sterile water, and diluted to provide suspensions of 10^7^, 10^6^, 10^5^, and 10^4^ cells/mL. Aliquots of 5 µL for each suspension were inoculated onto a solid YPD medium containing appropriate concentrations of allyl alcohol (AA; 0.05 and 0.1 mM), pyrazole (PYR; 0.1 mM), and/or disulfiram (DSF; 0.01 mM). Freshly prepared stock solutions of AA (100 mM stock in sterile water), PYR (100 mM stock in sterile water), or DSF (10 mM stock in absolute ethanol) was added to sterile media after cooling to approximately 55 °C. Colony growth was inspected after 48 h.

### 4.4. Protein Extraction

The cells from the exponential phase of the culture were centrifuged, washed twice with MilliQ water, and suspended in a cold homogenization buffer (20 mM phosphate buffer, pH 6.8, containing 1 mM EDTA and 1 mM PMSF). Then, the biomass was disrupted with 0.5 mm glass beads in 6 cycles of 30 s with intervals for cooling the sample on ice, and then centrifugation (14,000× *g*, 15 min, 4 °C). Supernatants were transferred to fresh tubes and immediately frozen at −80 °C. Protein concentration was determined using the Coomassie Protein Assay Reagent (Thermo Scientific, Waltham, MA, USA).

### 4.5. Enzyme Assays

Alcohol dehydrogenase (ADH; EC 1.1.1.1) activity in whole-cell protein extracts was determined by measuring the rate of NAD^+^ (0.48 mM) reduction at 340 nm with allyl alcohol or ethanol (8 mM) as a substrate in a 0.1 M sodium glycine buffer with pH 8.8 at room temperature (25 °C) [56] using a Varian Cary50 spectrophotometer. The ADH activity was expressed as U per mg of protein.

Aldehyde dehydrogenase (ALD; EC 1.2.1) activity was determined with an Alcohol Dehydrogenase Activity Colorimetric Assay Kit (Sigma-Aldrich) according to the manufacturer’s protocol with its own modification. In this assay, acetaldehyde was oxidized by ALD generating NADH, which reacts with a probe producing a colorimetric product proportional to the ALD activity present in the whole-cell protein extracts. The absorbance was measured against the blank for 36 min every 2 min using a Tecan Infinite M200 microplate reader (Tecan Group Ltd., Männedorf, Switzerland) at 450 nm. Alternatively, aldehyde dehydrogenase activity was determined with 6-methoxy-2-naphthaldehyde (MONAL-62) as a fluorogenic substrate in the presence of NAD^+^ [57]. The fluorescence signal increase was recorded for 7 min using a Tecan Infinite M200 microplate reader at λ_ex_ = 310 nm and λ_em_ = 360 nm. The ALD activity was expressed as U per mg of protein in both cases.

The protein samples (20 or 40 µg/path) were also separated by the native PAGE (Bio-Rad, Hercules, CA, USA). After electrophoresis, zymographic analyses were performed to reveal the activities of either alcohol or aldehyde dehydrogenases. The reagent solution for enzymatic staining contained 25 mg of NAD^+^, 15 mg of *p*-nitroblue tetrazolium chloride (NBT), 1 mg of phenazine methosulfate (PMS), 5 mL of 100 mM allyl alcohol, 10 mL of 25 mM Tris-HCl buffer with pH 7.5, and 31 mL of distilled water. Assays in the presence of ethanol, acetaldehyde, and acrolein as reaction substrates, as well as with NADH as a reaction cofactor, were also performed. Analyses of gel the images were performed using Azure Analysis Software.

The total activity of PP pathway dehydrogenases (assumed to be a sum of both ZWF1 and GND1/GND2 activities) was determined with D-glucose-6-phosphate and 6-phosphogluconate as substrates, and 6-phosphogluconate dehydrogenase (GND1 and GND2; EC 1.1.1.44) activity, was determined with 6-phosphogluconate, in the presence of NADP^+^ in both cases [58,59]. The kinetics of absorbance increase was recorded for 3 min using a Tecan Infinite M200 microplate reader at 340 nm. Glucose-6-phosphate dehydrogenase (ZWF1; EC 1.1.1.49) activity was calculated by subtracting the activity of GND1 and GND2 from the total enzyme activity. The data were expressed as arbitrary absorption units.

### 4.6. Western Blot

The protein samples were separated by SDS-PAGE and then transferred to a nitrocellulose membrane (PVDF Western Blotting Membranes, Roche, Basel, Switzerland) by semidry immunoblotting (Bio-Rad). The presence of proteins on the membrane was confirmed by Ponceau S (Sigma-Aldrich) labeling. After blocking with a PBST buffer (PBS, 0.1% Tween 20) containing 3% nonfat milk, the following primary antibodies were used: anti-yeast alcohol dehydrogenase (1:5000, ab34680, Abcam, Cambridge, UK), anti-yeast aldehyde dehydrogenase (1:5000, ab182893, Abcam), anti-glucose-6-phosphate dehydrogenase (1:2000, ab87230, Abcam), anti-6-phosphogluconate dehydrogenase (1:2000, ab125863, Abcam), and anti-β-actin (1:5000, MA5-15739, Thermo Fisher Scientific). The respective proteins were detected after incubation with the horseradish peroxidase-conjugated secondary antibodies (1:10,000, 111,035,003, Jackson ImmunoResearch or 1:10,000, AP160P, Millipore, Merck, Rahway, NJ, USA) with a SuperSignal West PICO Chemiluminescent Substrate (Pierce Biotechnology, Waltham, MA, USA), according to the manufacturer’s protocol. The images were captured using an Imaging Systems c300 (Azure Biosystems, Inc., Dublin, CA, USA).

### 4.7. Two-Dimensional Gel Electrophoresis, Mass Spectrometry and Protein Identification

The protein samples were separated by two-dimensional gel electrophoresis. In the isoelectrofocusing step (IEF, the first dimension), the whole-cell protein extracts were loaded onto 7 cm IPG strips (Bio-Rad) with a pI ranging from 3 to 10. The strips were rehydrated passively for 12 h at 20 °C, followed by an isoelectrofocusing run using the Protean IEF Cell. Prior to the SDS-PAGE, the IPG strips were equilibrated in the buffer containing 6 M urea, 75 mM Tris-HCl, pH 8.8, 30% glycerol, and 2% SDS: in the presence of 1% DTT (10 min) and then in the presence of 2.5% iodoacetamide (10 min). The SDS-PAGE step (the second dimension) was carried out according to Laemmli [60] using Protean II xi Cell 16 × 16 cm slab unit (Bio-Rad). In order to maximize the reproducibility of spot patterns and optimize the matching of protein profiles, both IPG strips obtained upon IEF of the WT strain; the Δ*sod1* mutants were placed next to each other onto one SDS-polyacrylamide gel (the “two-in-one gel” technique) and was then overlaid with low melting point agarose (ReadyPrep overlay agarose, Bio-Rad). Proteins were detected with silver staining, according to Jungblut and Seifert [61]. The resultant gel images were analyzed with Azure Analysis Software. To enable efficient MS analysis, an additional, independent electrophoretic run was carried out, followed by gel staining without glutaraldehyde. Protein spots were excised from the gel and analyzed by liquid chromatography coupled to the mass spectrometer in the Laboratory of Mass Spectrometry, Institute of Biochemistry and Biophysics, Polish Academy of Sciences (Warsaw, Poland). Raw data files were pre-processed with Mascot Distiller software (ver. 2.4.2.0., Matrix Science, Boston, MA, USA). The obtained peptide masses and fragmentation spectra were matched to the *Saccharomyces cerevisiae* Genome Database using the Mascot search engine (Mascot Daemon ver. 2.4.0, Mascot Server ver. 2.4.1, Matrix Science).

### 4.8. Determination of Tryptophan Content

The tryptophan content in the whole-cell protein extracts (0.1 mg/mL) was estimated as described previously [62] using a Tecan Infinite M200 microplate reader at λ_ex_ = 290 nm and λ_em_ = 325 nm. The results were expressed in arbitrary units.

### 4.9. RNA Samples

RNA samples were obtained using GeneMATRIX Universal RNA Purification Kit according to the manufacturer’s protocol (EURx, Gdańsk, Poland). Cells from the exponential phase of the culture (5 × 10^7^ cells/mL) were centrifuged, washed twice with MilliQ water, and suspended in the spheroplast buffer (1 M sorbitol, 0.1 M EDTA, 0.1% β-mercaptoethanol) containing lyticase (250 U per sample) for 30 min at 30 °C. The resultant spheroplasts were used for RNA isolation. RNA samples were stored at −20 °C, and each of them was thawed only once. The concentration and purity of RNA samples were measured with a Tecan Infinite M200 reader equipped with a NanoQuant Plate using a 260 nm/280 nm ratio.

### 4.10. Real-Time PCR

A total of 500 ng of RNA previously treated with DNase I (Roche) for 60 min, 25 °C (10 U per 1 µg RNA) was used for reverse transcription. To synthesize cDNA, SuperScript III First-Strand Synthesis SuperMix (Invitrogen, Thermo Fisher Scientific) was applied according to the manufacturer’s protocol, and the samples were stored at −20 °C until use. Real-time PCR was performed using 7500 Fast System (Applied Biosystems, Waltham, MA, USA) equipment and TaqMan chemistry. Briefly, the cDNA sample was diluted and mixed with TaqMan Gene Expression Master Mix and TaqMan Gene Expression Assays (Applied Biosystems, Life Technologies); at the same time, a PCR reaction was run in triplicate. The *ADH1*, *ADH2*, *ADH5*, *ALD2*, *ALD3*, *ALD6*, *ZWF1,* and *GND1* genes expression level were tested. *ACT1* was used as an internal control gene. The relative gene expression was calculated with a comparative C_T_ method: the −ΔΔC_T_ method for comparison of individual gene expression between the WT and Δ*sod1* mutant or −ΔC_T_ for multiple gene expressions in the same strain [63].

### 4.11. Determination of NAD(H) and NADP(H) Content

NAD(H) and NADP(H) content in the yeast cells was assessed with NAD/NADH-Glo Assay and NADP/NADPH-Glo Assay kits (Promega, Madison, WI, USA) according to the manufacturer’s protocols with our own modifications [59]. Cells from the exponential phase of the culture were centrifuged, washed, suspended to a density of 2 × 10^6^ cells/mL in a PBS buffer, and then subjected to the determination of cofactors. Luminescence was recorded for 3 h using a Tecan Infinite M200 microplate reader. The value of the blank was subtracted each time. The results were presented as individual and total pyridine cofactors content. In addition, the (NADPH/NADP^+^)/(NADH/NAD^+^) ratio was calculated.

### 4.12. Statistical Analysis

The results are presented as mean ± SD from at least three independent experiments. The statistical analysis was performed using the IBM SPSS 21.0 software. The statistical significance of differences between the WT strain and Δ*sod1* mutant was evaluated with a *t*-test. The differences between the expression of various genes were compared using one-way ANOVA and Tukey post hoc test. The homogeneity of variance was checked to employ Levene’s test. The values were considered significant at *p* < 0.05.

## Data Availability

Not applicable.

## Supplementary Materials

The following supporting information can be downloaded at: https://www.mdpi.com/article/10.3390/ijms24010659/s1.
