# Supplementary material for: Unbalance between Pyridine Nucleotide Cofactors in The SOD1 Deficient Yeast Saccharomyces cerevisiae Causes Hypersensitivity to Alcohols and Aldehydes
Source: Int J Mol Sci. 2022 Dec 30;24(1):659. doi: 10.3390/ijms24010659 (PMC9820918; doi:10.3390/ijms24010659)
Supplement: Supplementary file 1 [file ijms-24-00659-s001.zip › ijms-2101902-supplementary.pdf]

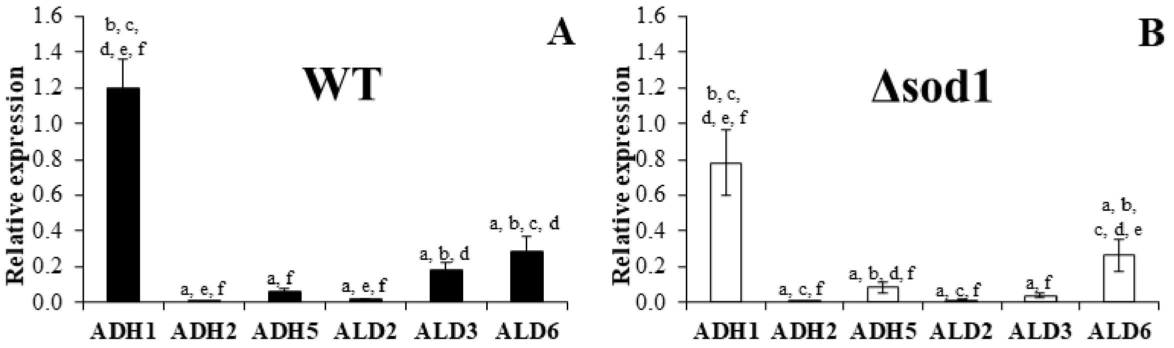

Figure S1. *ADH* and *ALD* genes expression in the wild-type (WT) strain (**A**) and Δ*sod1* mutant (**B**). *ADH* and *ALD* genes expression were performed by qPCR assay with TaqMan probes. The relative gene expression was calculated with a comparative C_T method: $-\Delta C_T$ for multiple gene expression in the same strain. The results are presented as mean ± SD from three independent experiments; a, with respect to *ADH1*; b, with respect to *ADH2*; c, with respect to *ADH5*; d, with respect to *ALD2*; e, with respect to *ALD3*; f, with respect to *ALD6*.
